# Cohesive Shred Gold

**Published:** 1867-05

**Authors:** E. Lamm


### COHESIVE SHRED GOLD.

INVENTED AND MANUFACTURED BY E. LAMM.

This preparation of gold for filling teeth is very similar to some of the preparations of crystal gold made years ago. When well prepared it has some advantages over any other form of gold, for filling teeth. For instance, in laying a foundation in large cavities, it is preferable to anything else, as by proper manipulation, it takes a firm hold upon the dentine, and will remain firmly in position, and serve as a good attachment for the remaining part of the filling; with it, too, large cavities can be much more rapidly filled than by foil in the ordinary method of using. Skill and care are necessary in using it. Another advantage claimed for it is that it can be efficiently used when moist, or even under water. We would make the suggestion here, however, that it is not best to make this too prominent, for such things are often taken advantage of, to the great detriment of operations. It is always safer to keep fillings free from moisture while being made, whatever material may be used, and we should feel better pleased by the introduction and adoption of some method of excluding the moisture, rather than to work in it, with any material.

This gold is for sale by S. S. White, and we presume by the Depots generally.

